# Intraoperative dexamethasone on left atrial function and postoperative atrial fibrillation in cardiac surgical patients

**DOI:** 10.1186/cc13372

**Published:** 2014-03-17

**Authors:** K Jacob, S Dieleman, H Nathoe, D Van Osch, E De Waal, M Cramer, J Kluin, D Van Dijk

**Affiliations:** 1 Utrecht University Medical Center, Utrecht, the Netherlands

## Introduction

Postoperative new-onset atrial fibrillation (PNAF) is very common after cardiac surgery. The inflammatory response due to surgery and cardiopulmonary bypass (CPB) may contribute to PNAF by inducing atrial dysfunction [1]. Corticosteroids reduce the inflammatory response and may thus reduce atrial dysfunction and PNAF [2]. The aim of this study was to determine whether dexamethasone protects from left atrial dysfunction and PNAF in cardiac surgical patients.

## Methods

Patients undergoing cardiac surgery were randomized to a single dose of dexamethasone (1 mg/kg) or placebo after inducing anesthesia. Transesophageal echocardiography was performed in patients after CPB. The primary outcome was left atrial total ejection fraction (LA-TEF) after sternal closure; secondary outcomes included left atrial diameter and PNAF, detected by Holter monitoring.

## Results

**Figure 1:**
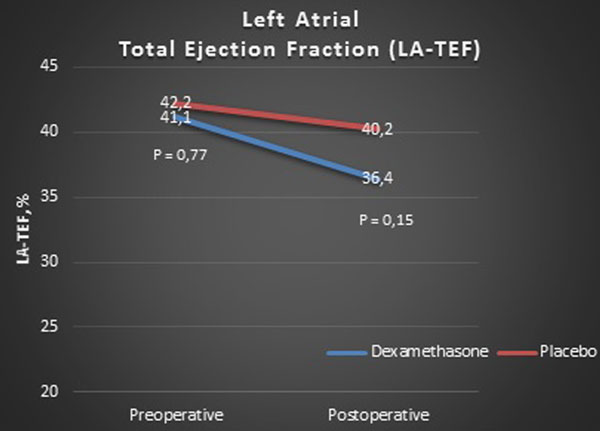
**Primary outcome preoperatively and postoperatively in dexamethasone and placebo groups**.

Sixty-two patients were included. Baseline characteristics were well balanced. Postoperative LA-TEF was 36.4% in the dexamethasone group and 40.2% in the placebo group (*P *= 0.15) (Figure 1). Secondary echocardiographic outcomes were also insignificant (Table 1). The incidence of PNAF was 30% in the dexamethasone group and 39% in the placebo group (*P *= 0.47).

**Table 1:** Secondary postoperative echocardiographic parameters in both groups

Parameter	Dexamethasone	Placebo	*P ***value**
LA-TEF	36.4	40.2	0.15
LA diameter	4.6	4.3	0.19
LA area	16.0	16.4	0.81

## Conclusion

Intraoperative high-dose dexamethasone did not have any protective effect on postoperative LA-TEF or dimension and did not reduce the risk of PNAf in cardiac surgical patients.
